# DNA methylation contributes to plant acclimation to naturally fluctuating light

**DOI:** 10.1111/nph.70567

**Published:** 2025-09-20

**Authors:** Robyn A. Emmerson, Philip Davey, Mouesanao Kandjoze, Ulrike Bechtold, Nicolae Radu Zabet, Tracy Lawson

**Affiliations:** ^1^ School of Life Sciences University of Essex Colchester CO4 3SQ UK; ^2^ Blizard Institute, Barts and The London School of Medicine and Dentistry Queen Mary University of London London E1 2AT UK

**Keywords:** *Arabidopsis thaliana*, DNA methylation, environment, epigenetics, fluctuating light, RNA‐seq, transposable elements

## Abstract

- Plants in the natural environment experience continuous dynamic changes in light intensity. Here, we exposed *Arabidopsis thaliana* plants to naturally fluctuating light (FL) regimes alongside traditional square light (SQ) regimes such as those often found in control environment growth chambers.
- The physiological response was highly consistent across experiments in sibling plants, indicating the possibility of an epigenetic mechanism, leading us to investigate differences in DNA methylation.
- Our results identified a large number of changes in DNA methylation patterns between FL‐acclimated plants and SQ‐acclimated plants, demonstrating that natural fluctuations in light impact plant epigenetic mechanisms. Most importantly, there are more differences in DNA methylation patterns between different light pattern regimes than between different light intensities. These differences in DNA methylation were accompanied by significant changes in gene expression, some of which correlated with altered DNA methylation. One of these genes, MCCA, was found to significantly impact photosynthetic efficiency when knocked out. Thousands of transposable element (TE) copies were differentially methylated between light regimes. Interestingly, up to 30% of these TEs are linked to nearby differentially expressed genes.
- Our data suggest DNA methylation plays a role in acclimation to natural light, which may directly regulate gene expression and impact TE activation.

## Introduction

In natural environments, plants are continually exposed to changing environmental conditions, including rapidly altering light intensity due to weather and shading from overlapping leaves, as well as diurnal and seasonal effects (Botta *et al*., 2000; Berry & Smith, 2012; Masson *et al*., 2013). Light is crucial for photosynthesis, and variations in its quantity, quality, and timing significantly impact plant growth, development, and productivity (Kami *et al*., 2010; Zhang *et al*., 2011; Bayat *et al*., 2018). To manage these potentially stressful fluctuations, plants undergo acclimation, a process involving alterations in molecular physiology. Although numerous studies have examined the impact of light intensity, the mechanisms for acclimation to dynamic irradiance have received limited attention to date.

Acclimatory responses have been correlated with epigenetic changes as both a response to the stress (Grativol *et al*., 2012; Sahu *et al*., 2013; Crisp *et al*., 2016; Thiebaut *et al*., 2019) and a priming effect (Hilker & Schmülling, 2019; Godwin & Farrona, 2020). The dynamic nature of DNA methylation and its potential to control gene expression has previously been studied in plant stress responses and acclimation (Liu & He, 2020; Saeed *et al*., 2022) as it represents a fast and changeable mechanism by which plants can respond to their environments.

Nevertheless, there is currently limited evidence for the role of DNA methylation in light stress responses and acclimation. Short‐term light stress, with a small number of fluctuations in light, has been reported to have limited effects on DNA methylation (Ganguly *et al*., 2018). However, production of reactive oxygen species (ROS), including hydrogen peroxide (H_2_O_2_), as a response to high light has been linked to alterations to the methylome. In tobacco mutants, which overproduce H_2_O_2_, loss of DNA methylation was observed in comparison with the control plants (Villagómez‐Aranda *et al*., 2021), suggesting overproduction of H_2_O_2_ correlates with loss of methylation.

More generally, ROS have been implicated in epigenetic reprogramming. For example, MutS Homologue 1 (MSH1) present in sensory chloroplasts has been implicated in alterations to DNA methylation. Knockout of MSH1 results in genome‐wide reprogramming of DNA methylation (Virdi *et al*., 2015), further suggesting a role for chloroplast signalling in epigenetic change. Combined with knowledge that high‐light stress results in ROS production (Edreva, 2005; Pospíšil, 2016), this indicates that light environments can impact the plant methylome. There is also evidence that small RNAs (sRNAs) are induced in response to high light in *Arabidopsis* and impact gene expression (Tiwari *et al*., 2021). Since 24‐nt sRNAs are capable of guiding DNA methylation via the RNA‐directed DNA methylation (RdDM) pathway (Lewsey *et al*., 2016), this suggests another possible mechanism by which the light environment can impact DNA methylation.

**Fig. 1:**
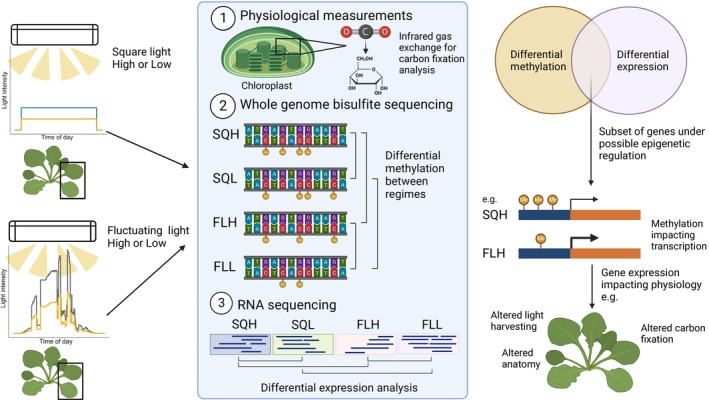
Schematic representation of the experimental design used in this study. *Arabidopsis thaliana* (Col‐0) were exposed to one of four light regimes, two representing laboratory light conditions of high and low intensity (square light high and square light low), and two representing natural light conditions (fluctuating light high and fluctuating light low). These were then subjected to physiological assessment, followed by DNA methylation (WGBS‐seq) and transcriptome (RNA‐seq) analysis to determine whether the light regime impacted the epigenetic landscape, and whether there was a correlation between DNA methylation and gene expression that could be associated with the altered physiology. This figure was created in BioRender (BioRender.com/cgni940).

Although these studies investigate excess light stress or peaks and troughs in light, none are reflective of environmental light regimes in which the fluctuating frequency is much greater. Our previous work showed that the physiology of *Arabidopsis* is impacted by a naturally fluctuating light (FL) regime (Vialet‐Chabrand *et al*., 2017; Matthews *et al*., 2018). Here, we have utilized naturally FL regimes (Vialet‐Chabrand *et al*., 2017) to assess the impact of acclimation on DNA methylation and gene expression in *Arabidopsis thaliana* (Fig. 1). Our results showed large differences in DNA methylation patterns of plants exposed to FL regimes compared with plants exposed to square wave light. In particular, we found hundreds of differentially methylated regions (DMRs) in CpG and non‐CpG context, and these regions consist of both genes and transposable elements (TEs). Furthermore, we linked these epigenetic changes to changes in transcription that were able to explain the observed phenotypes.

## Materials and Methods

### Experimental model and growth conditions

*Arabidopsis thaliana* (L.) Heynh. Col‐0 background or *mcca* (SALK_137966C) were collected from a single parent, reducing the likelihood of genetic differences, and germinated in 5‐cm^2^ pots on a peat‐based compost (Levingtons F2S, Everris, Ipswich, UK), and placed in a controlled growth environment at 65% relative humidity, 8 h : 16 h, 22°C, light : dark cycle, CO_2_ concentration 400 μmol mol^−1^. At 14 d, the seedlings were transplanted to individual 5‐cm^3^ pots containing the same soil as described previously and returned to the controlled environment.

At the 4‐leaf stage, plants were removed from the controlled environment and placed under Heliospectra LED light source (Heliospectra, Göteborg, Sweden) programmed to each light regime (Supporting Information Fig. S1) in a dark room maintained at 21°C : 16°C day : night, 50% relative humidity. Average light intensity for high‐light conditions was 460 and 230 μmol m^−2^ s^−1^ for low‐light conditions on a 12 h : 12 h, day : night cycle. Plants were kept in well‐watered conditions, with their position under the light source randomized every 3 d to remove any potential heterogeneity in spectral quantity and quality. Light regimes are available in Table S1.

### Physiological measurements

After 20‐d acclimating to the light regimes, plants were subjected to infrared gas exchange analysis. The newest fully expanded leaf was placed in the measuring cuvette of a LiCor 6800 and photosynthesis assessed as a function of internal carbon dioxide concentration (*C*_i_) and as a function of light intensity.

Chl fluorescence assessment of *met1‐1* mutants was carried out using a CFImaging system (Technologica Ltd, Colchester, UK). After 7 d of acclimation, four plants (one from each group) were analysed at a time and exposed to a light–response curve protocol of decreasing light steps starting at 1500 μmol m^−2^ s^−1^.

### DNA extraction – CTAB method

The three newest fully expanded leaves were harvested between 12 pm and 12:30 pm (Zeitgeber time 5–5.5) on Days 18–21 of vegetative growth and flash‐frozen (*n* = 6) in the following order: square light high (SQH), square light low (SQL), fluctuating light high (FLH), and fluctuating light low (FLL). To minimize the effect of the time of sampling on the results, all tissues were harvested within only a few minutes of each other. A modified version of the cetyltrimethylammonium bromide (CTAB extraction protocol described by Porebski *et al*. (1997) was utilized.

### Whole genome bisulfite sequencing

Five microliters of genomic DNA from multiple samples per regime (*n* = 6 plants/regime) was pooled together (total volume 30 μl) to form a single sample. Library preparation and sequencing were carried out by Novogene.

DMRs were computed with DMRcaller (Catoni *et al*., 2018) using the bins method, with a bin size of 150 base pairs as done previously (Hansen *et al*., 2012; Catoni *et al*., 2017). A *P*‐value threshold of 0.01 for all contexts was used, alongside a minimum cytosine count of 4, a minimum proportion difference of 0.2 for CpG and CpHpG, and 0.1 for CpHpH, and a minimum reads per cytosine of 4, allowing bins with few cytosine bases to be avoided (Stroud *et al*., 2013). Statistics for preprocessing of whole‐genome bisulfite sequencing (WGBS) datasets are included in Table S2.

For assessing the chromatin state of differentially methylated TEs, data on chromatin states were taken from Jamge *et al*. (2023) and overlapped with our data using findOverlaps from the R package GenomicRanges (Lawrence *et al*., 2013).

### RNA extraction and sequencing

The RNA from three independent replicates (100 mg per replicate) per regime was extracted using the Macherey‐Nagel Mini kit for RNA purification (Macherey‐Nagel, Allentown, PA, USA). Sample purity was assessed using the NanoDrop ND‐1000 Spectrophotometer (NanoDrop; ThermoFischer, Wilmington, DE, USA). Library preparation (poly‐A) and sequencing were carried out by Novogene. Sequencing was performed on the Illumina NovaSeq 6000 (Illumina, San Diego, CA, USA), utilizing a paired end 150‐bp read length, with 6‐Gb raw data generated per sample.

Raw data files were aligned to TAIR10 (Howe *et al*., 2019) using Hisat2 (Kim *et al*., 2019), and DESeq2 (Love *et al*., 2014) was used to detect differentially expressed genes (DEGs) (*P*‐adjusted value ≤ 0.05, log_2_FC ≥ 0.5). Statistics for preprocessing of RNA‐seq datasets are included in Table S3.

For assessment of diurnal expression patterns, our data were overlapped with count files from Redmond *et al*. (2025) using findOverlaps.

## Results

### Growth light regime impacts DNA methylation

To investigate the impact of the growth light regime on DNA methylation, plants were acclimated to SQ and FL regimes for 21 d. In both cases, we considered high and low light, which resulted in four conditions: SQH, SQL, FLH, and FLL (Fig. 1). SQ refers to regimes in which the light comes on at one light level and maintains this until the end of the day, when the light is turned off; the light is either off or on with no variance in the light intensity throughout the day. For the FL regimes, light mimics a natural pattern, with gradual increases in intensity at dawn and dusk and peaks and troughs in light intensity throughout the day, peaking at the middle of the day cycle. Importantly, each pair of SQ and FL regimes provides the same amount of light over the 12‐h period; it is only the way in which it is provided that differs. Additionally, the FLL regime was created by halving the FLH regime, allowing a lower light level to be provided while still mimicking a natural regime.

The plants were then subjected to physiological assessment (Fig. S1) to ensure a consistent phenotype with previous data (Vialet‐Chabrand *et al*., 2017). In short, plants acclimated to SQ demonstrated a significantly higher level (*P* < 0.05) of carbon fixation as a function of changing internal carbon dioxide than FL‐acclimated plants, with plants under lower light demonstrating significantly lower levels than their high‐light counterpart. However, under increasing light levels, FLH plants show a significantly higher (*P* < 0.05) level of assimilation than all other conditions at light levels above 250 μmol m^−2^ s^−1^. Plants acclimated to lower light intensities again demonstrated lower levels of assimilation, while FLL plants were significantly lower (*P* < 0.05) than SQL plants. This suggests that light acclimation impacts the biochemical and operational responses differently depending on growth light regime, with possible lower biochemical efficiency seen in plants acclimated to FL than those under SQ.

**Fig. 2:**
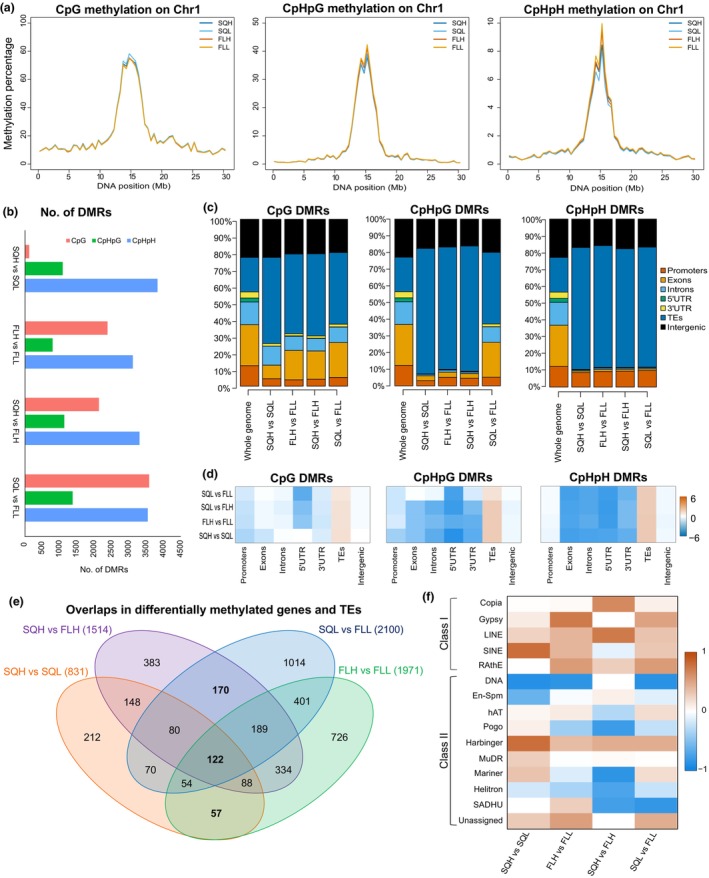
The effects of light regime on DNA methylation in *Arabidopsis thaliana*. (a) Low‐resolution profiles of DNA methylation in the four light conditions. We considered separately CpG, CpHpG, and CpHpH (where H is A, C, or T) methylation patterns and only plot methylation data on Chromosome 1. (b) Number of differentially methylated regions (DMRs) between the different conditions. (c) Annotation of the DMRs to different genomic features (promoters, exons, introns, 5′ untranslated regions (UTRs), 3′UTRs, transposable elements (TEs), and intergenic regions), and (d) enrichment computed as log_2_(observed/expected) for the genomic features. (e) Venn diagram of the overlaps of the genes and TEs that display differential methylation between the four light regimes. (f) TEs subfamilies and evaluation of the change in DNA methylation. TEs that gain methylation (1) are marked by red and TEs that lose methylation (−1) are marked by blue, calculated as log_2_[observed]/[expected] DMRs. SQH, SQL – square light regime of high or low intensity, respectively. FLH, FLL – fluctuating light regime of high or low intensity, respectively.

DNA from six independent replicates from each regime was pooled to form a single sample per regime. Note that it has been shown previously that a single replicate can recover *c*. 86% of DMRs compared with experiments using multiple biological replicates (Catoni *et al*., 2018). Our results showed that there is some change in the global methylation profiles between the four growth conditions, particularly within the centromeric and pericentromeric regions (Figs 2a, S2), although little variation is noted in the chromosome arms in which coding genes are located. Together, this indicates that light acclimation is likely not resulting in global changes in the epigenetic machinery, but instead may be impacting DNA methylation at specific regions in the genome.

Next, we computed the DMRs between SQ and FL regimes and identified between 119 and 3574 DMRs in CpG, 793 and 1373 DMRs in CpHpG, and 3105 and 3814 DMRs in CpHpH contexts (Figs 2b, S3; Table S4). This is markedly higher than previous reports in which plants were acclimated for 7–8 d (Ganguly *et al*., 2018), which indicates that the level of epigenetic changes might be impacted by the duration of the acclimation to the FL regime. Interestingly, we found that more epigenetic changes are observed between SQ and FL regimes than between low and high light. This suggests that plants display a stronger acclimation when changing the light frequency/pattern than to changes in the amplitude of the light. Some of these regions gain DNA methylation, while others lose it, but there is no clear trend for that in the four comparisons we performed (Fig. S3).

Functional annotation of these DMRs indicates that acclimation could be linked to epigenetic changes in TEs and intergenic regions (Fig. 2c,d). In some specific cases, we also found a high number of DMRs in introns and promoters. These, together with intergenic regions, are potential regulatory regions, indicating they are involved in gene regulation. TEs accounted for most DMRs across all contexts, indicating that the epigenetic state of TEs could contribute during acclimation to all growth light regimes. Most importantly, and as expected, if we consider all three methylation contexts, DMRs are only enriched at TEs; that is, there are more DMRs within TEs than expected by chance (note the positive values for log_2_[observed/expected] in Fig. 2d).

Together, our data suggest that DNA methylation profiles changed in response to different growth light regimes, with differences between the light frequency resulting in a higher number of DMRs compared with differences in light intensity.

### Exposure to fluctuating light regimes leads to hypermethylation of transposable elements

Next, we investigated whether there are common changes in DNA methylation at genes and TEs occurring between the four different light regimes (Fig. 2e). Our results showed that most of the DMRs within genes or TEs were unique to the light regime, suggesting that there is a distinct epigenetic change associated with acclimation to the growth light regimes. The highest number of unique DMRs was noted in SQL vs FLL, suggesting a greater degree of regulation may be required under fluctuating low‐light conditions. One hundred and twenty‐two genes and TEs (called light constitutive features) were differentially methylated across all regimes, indicating that these may be essential to growth light acclimation, regardless of the actual light regime (Fig. 2e; Table S5). Furthermore, 170 differentially methylated genes and TEs (called light frequency features) were shared between SQHvFLH and SQLvFLL independent of the light intensity and were not identified between SQHvSQL and FLHvFLL. Finally, 57 differentially methylated genes and TEs (called light intensity features) were shared between hSQHvSQL and FLHvFLL, independent of the light frequency, but not between SQvFL regimes. Of the 122 light constitutive features, the majority (90) were TEs (Table S5), meaning TE regulation could have a role in the acclimation response. Gene functions included Vacuolar H+ ATPase (AT3G58730), as well as proteins associated with signalling in the chloroplast (AT1G19090) and leaf senescence (AT1G54040). When the overrepresentation analysis of these groups was considered, no functional groups were found to be significantly enriched (false discovery rate < 0.05).

Fig. 2(c) showed that a large proportion of DMRs between different light regimes overlapped TEs. To further investigate this, we considered different types of TEs and found that DMRs were enriched in the majority of retrotransposons (Class I TEs) and depleted in DNA transposons (Class II) (Fig. 2f), with enrichment referring to the ratio of differential methylation observed divided by the expected from random sampling of methylation across the genome. For example, long interspersed nuclear elements (LINE) TEs were enriched across all regimes, while Copia were only enriched in DMRs between SQ and FL, independent of the light intensity. Gypsy and short interspersed nuclear element (SINE) TEs were enriched in all cases except between SQH and FLH high. This indicates that acclimation to light can result in changes in DNA methylation at specific classes of retroelements.

### Epigenetic changes upon exposure to light regimes correspond to activation in transcription

**Fig. 3:**
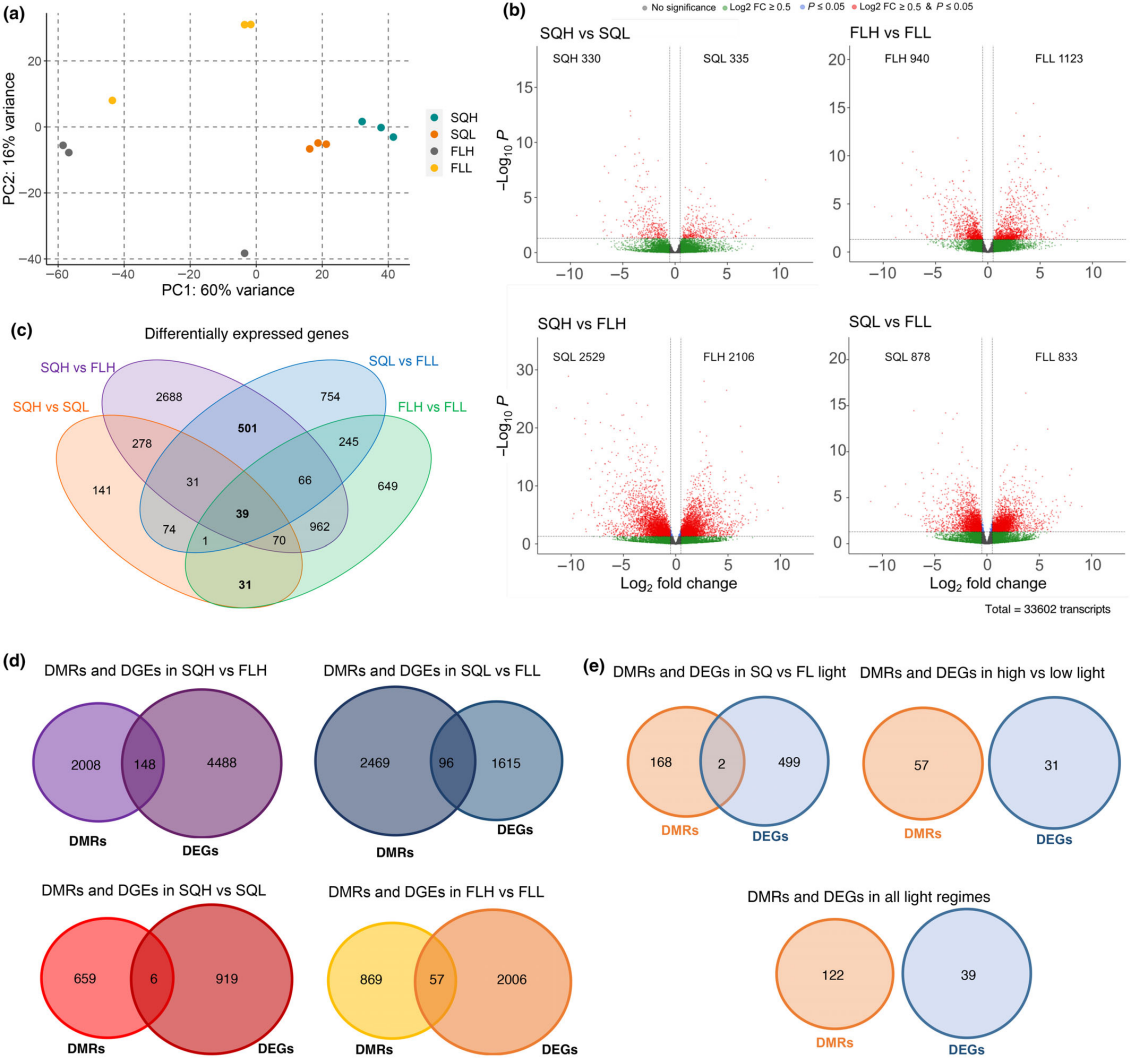
Transcriptome of *Arabidopsis thaliana* plants exposed to the different light regimes. (a) Principal Component Analysis of RNA‐seq data in three biological replicates for the four light conditions. (b) Volcano plots for the significantly differentially expressed genes (DEGs). The plot highlights the genes that show differential expression between the two light regimes in the header using a *P*‐value threshold of 0.5 and a log_2_ fold change threshold of 0.5. The number in the corresponding inset represents the number of genes with higher expression in the corresponding light regime. (c) Venn diagram identifying common and specific sets of genes differentially expressed in the four comparisons. Five hundred and one genes are differentially expressed in fluctuating compared to square light regimes, 31 in high‐light regions compared to low‐light regions, and 39 between all comparisons. (d) Venn diagrams of genes that are differentially expressed in one of the four comparisons and also overlap with a differentially methylated region (DMR) in any context in the corresponding comparison. (e) Genes that are differentially expressed and overlap with DMRs in the three combined comparisons, namely: (i) fluctuating vs square light, (ii) high vs low intensity, and (iii) all comparisons. SQH, SQL – square light regime of high or low intensity, respectively. FLH, FLL – fluctuating light regime of high or low intensity, respectively.

To determine whether the DNA methylation is accompanied by transcriptional changes in the plants acclimated to FL, we performed RNA‐seq in three biological replicates for the four light regimes. Principal component analysis confirmed that the biological replicates grouped together (Fig. 3a). The groups of FLH and FLL clusters displayed a greater degree of spatial separation in comparison with SQH and SQL, indicating that gene expression displays higher variability in FL than in SQ regimes, even in the case when the FL pattern is the same for all three biological replicates (Fig. S4). Since all tissue was harvested within only a few minutes of each other, it is unlikely that the time of sampling impacted this. This would require further exploration to explain sufficiently.

Next, we investigated whether genes are differentially expressed between the four light regimes and found that between hundreds and thousands of genes are indeed differentially expressed genes (DEGs) (Fig. 3b; Table S6). The lowest number of DEGs (665) was identified between SQH and SQL, indicating that changing light intensity upon exposure to a SQ leads to the lowest number of transcriptional changes. By contrast, comparing SQH with FLH led to the highest number of DEGs (4635), which means that changes in light frequency had the largest effect on the plants. Overall, our results indicate that acclimation to FL requires a greater magnitude of change than acclimation to light intensity. To assess whether a subset of these genes is shared across regimes, DEGs were grouped depending on the regime (Fig. 3c). Thirty‐nine genes were found to be differentially expressed across all regime comparisons, indicating these genes may be key for light acclimation. A further 31 genes were shared between high‐ and low‐light intensity (SQH vs SQL and FLH vs FLL), and 501 genes were differentially expressed between SQ and FL (SQH vs FLH and SQL vs FLL). Gene ontology (GO) analysis revealed significant enrichment across nine terms in the 39 genes differentially expressed in all regimes (Fig. S5A), most of which were associated with stress responses, suggesting that the light regime significantly impacts how the plants respond to stress. Nevertheless, only the response to wounding term was enriched when comparing HL vs LL (Fig. S5B), which suggests that higher light intensity could potentially induce a response similar to wounding without necessarily the physical wounds. Interestingly, when comparing SQ vs FL conditions, we did not identify any stress response terms significantly enriched. However, metabolic and meiotic cell cycle terms were enriched when comparing SQ vs FL (Fig. S5C), suggesting metabolism and meiosis are primarily impacted in plants acclimated to light patterns.

DNA methylation is linked directly to transcriptional repression or gene silencing (Zhang *et al*., 2006; Zilberman *et al*., 2007), but this is not always the case, and it is now known that not all changes in DNA methylation translate into changes in gene expression (Lister *et al*., 2008; de Mendoza *et al*., 2022; Grant *et al*., 2024). To investigate whether the transcriptomic changes are linked with epigenetic state, we looked at genes that showed both differential expression and differential methylation in the different light regimes. Since the majority of our non‐TE DMRs are located within gene bodies or intergenic regions (Fig. 2c), we first performed the analysis with gene body DMRs (Fig. 3d,e; Table S7). Our results showed that there was a greater number of genes displaying both differential expression and differential methylation under SQH vs FLH and SQL vs FLL than under SQH vs SQL and FLH vs FLL (Fig. 3d). However, we cannot conclude that a statistically significant overlap between the different gene groups exists (chi‐squared, *P* > 0.9). The genes displaying differential expression that are under possible control of DNA methylation exhibit wide functionalities (Table S5), with many related to plastid activity and stress responses as well as photosynthesis‐related, demonstrating that light can have impacts on plant gene expression outside photosynthesis.

**Fig. 4:**
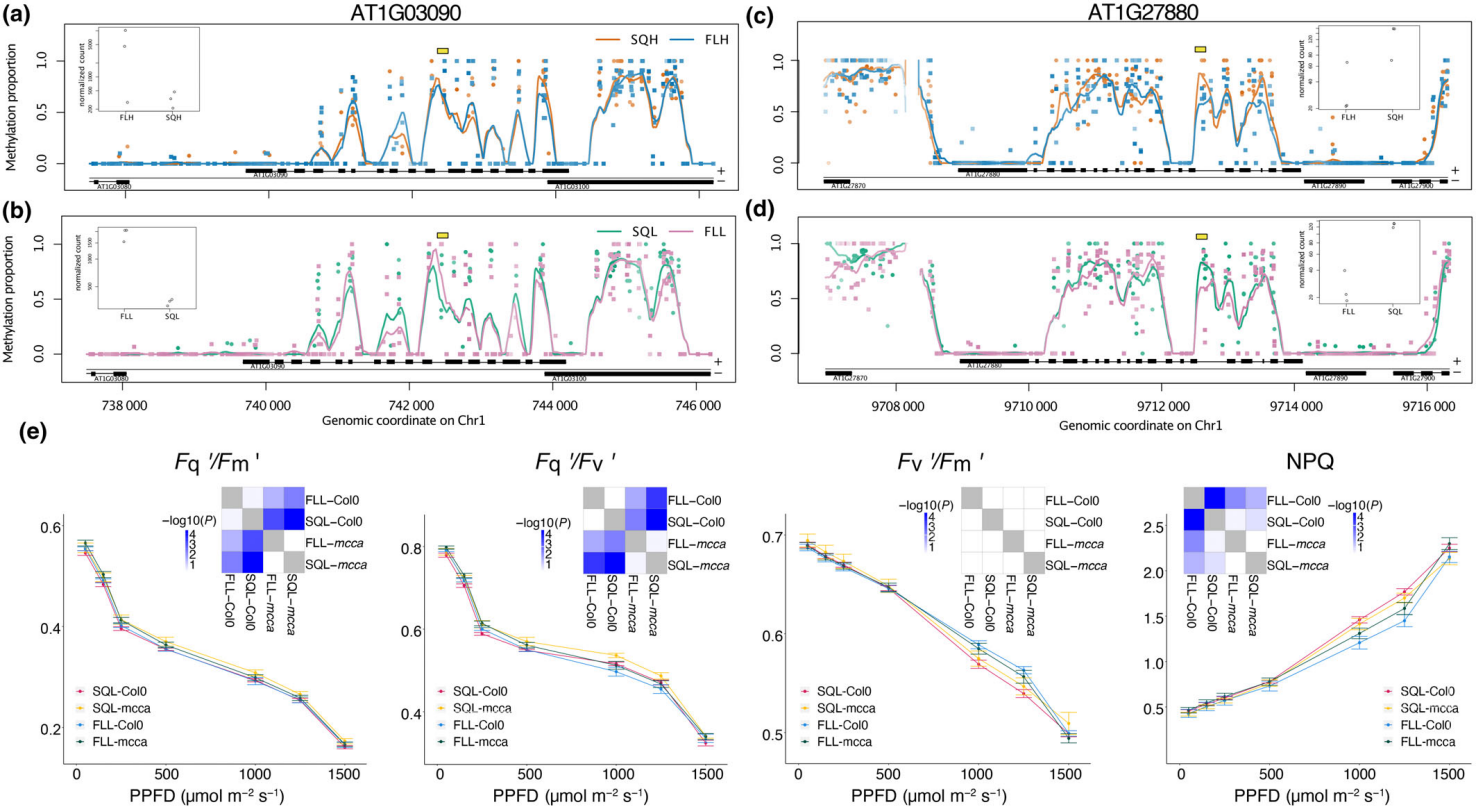
Methylation profiles of the two differentially methylated and differentially expressed genes (DEGs), and physiological validation of mcca in *Arabdiopsis thaliana*. We plotted: (a, b) MCCA and (c, d) AT1G27880. We considered separately the case of (a, c) square light high‐intensity (SQH) vs fluctuating light high‐intensity (FLH) and (b, d) the low‐intensity comparison (SQL vs FLL). The yellow rectangle at the top marks the DMR in the CpG context. (e) The effects of *mcca* under SQL vs FLL light on the operating efficiency of photosystem II (PSII) ($F_{q}^{\prime }/F_{m}^{\prime }$), photochemical quenching ($F_{q}^{\prime }/F_{v}^{\prime }$), maximum efficiency of PSII ($F_{v}^{\prime }/F_{m}^{\prime }$), and nonphotochemical quenching. *n* = 6, points represent the mean ± SE. Inlays show the −log_10_ of the *P*‐value (*post hoc* Tukey). SQH, SQL – square light regime of high or low intensity, respectively. FLH, FLL – fluctuating light regime of high or low intensity, respectively.

When SQ and FL regimes were grouped together, two genes were found to be both differentially methylated and expressed, but we cannot conclude that a statistically significant overlap between the different gene groups exists (chi‐squared, *P* > 0.9) (Figs 3e, 4a–d). This indicates that epigenetic variation does not contribute directly to changes in expression in most genes. However, the functions of these two genes suggest that there may be some level of involvement in the acclimatory response. Interestingly, the DMRs were located within introns of these genes, and the exact position is maintained independent of the light intensity; that is, the position of the DMR in SQH vs FLH is conserved in SQL vs FLL. This indicates a potential regulatory role of those regions, possibly as enhancers. One was AT1G03090 (MCCA), a subunit of 3‐methylcrotonyl‐CoA carboxylase (MCCase), which catalyses a key step in the catabolism of leucine and isovaleric acid in the mitochondria (Wurtele & Nikolau, 2000; Nikolau *et al*., 2003). Accumulation of MCCA is known to occur in response to darkness, while exogenous sucrose has been shown to decrease MCCA expression (Che *et al*., 2002), suggesting that there could be transcriptional control as a result of both light and sugar signalling. The second gene is AT1G27880, a DEAD/DEAH box RNA helicase family protein. Although this gene is not characterized, the general family contains a range of RNA helicases with specific expression patterns in different developmental stages (Xu *et al*., 2013). Expression of several family genes has been associated with abiotic stress tolerance, and reported to be induced by glucose, abscisic acid, and salt (Hsu *et al*., 2014; Matsumura *et al*., 2016).

To investigate whether the differential methylation and expression of *MCCA* contributes to the differences in physiology seen, the T‐DNA knockout mutant (*mcca*) was acclimated to SQL and FLL light, and Chl fluorescence imaging was conducted (Fig. 4e). In plants acclimated to SQL light, *mcca* exhibited significantly higher (*P* < 0.01) photosystem II (PSII) operating efficiency ($F_{q}^{\prime }/F_{m}^{\prime }$), although this was not seen under FLL. Both photochemical quenching ($F_{q}^{\prime }/F_{v}^{\prime }$) and the maximum efficiency of PSII ($F_{v}^{\prime }/F_{m}^{\prime }$) are mathematically related to $F_{q}^{\prime }/F_{m}^{\prime }$ (Murchie & Lawson, 2013), so changes in one of these measurements indicate which physiological differences may be differentially regulated in the mutant compared with the wild‐type (WT). SQL‐mcca had a significantly higher $F_{q}^{\prime }/F_{v}^{\prime }$ (*P* < 0.01) than Col‐0 plants, with no notable differences in the $F_{v}^{\prime }/F_{m}^{\prime }$. Under FLL light, a significantly higher $F_{q}^{\prime }/F_{v}^{\prime }$ was also seen (*P* < 0.03), with no significant difference in $F_{v}^{\prime }/F_{m}^{\prime }$. Nonphotochemical quenching (NPQ) estimates the heat loss from PSII (Murchie & Lawson, 2013) and represents the light energy that does not enter the photosystem. There was no significant difference in NPQ between SQL‐mcca and SQL‐Col0, but under FLL, NPQ was higher in the *mcca* plants than in Col‐0 (*P* < 0.01). Together, this suggests that MCCA expression may contribute to PSII efficiency and in regulating energy lost via NPQ. MCCA expression decreases between SQL and FLL (log_2_FC = −2.48; *P*‐adj = 0.029), thus suggesting a functional link between the differential methylation and expression between SQL and FLL‐acclimation and the physiological differences observed.

In addition, we investigated whether differential methylation within promoters of genes can be linked with differential expression and found very little overlap between the different conditions (Fig. S6). Our results showed that there is no gene that displayed differential methylation at promoters and differential expression that was common between SQ and FL regimes or between low‐light and high‐light regions.

Considerations of the impact of photoperiod and circadian regulation are important in the interpretation of this study. Recent evidence has shown that dawn and dusk act to alter regulatory patterns that are reminiscent of shorter photoperiods (Mehta *et al*., 2024). It is therefore possible that the differences in methylation and gene expression reported here are reflective of this, rather than the impact of FL relative to SQ. To investigate this, we took a published diurnal RNA‐seq dataset (Redmond *et al*., 2025) and overlapped this with our DEGs with DMRs across all regime comparisons. We found very few differentially methylated DEGs were differentially expressed across the diurnal period under all light regimes (Fig. S7), with only AT3G56940 and AT4G26850 displaying diurnal expression in SQvFL and only AT2G10940 displaying diurnal expression in HLvLL. Overall, this suggests that circadian regulation has little effect on the expression of these genes and that DNA methylation is likely not a circadian regulator in this instance.

### Changes in light regimes lead to transcription of TEs

Demethylation of TEs can lead to their activation (Miura *et al*., 2001; Slotkin *et al*., 2005; La *et al*., 2011; Saze *et al*., 2012; Benoit *et al*., 2019; Catoni *et al*., 2019). To determine whether the differential methylation has led to activation or silencing of TEs, we identified the set of TEs that were both differentially methylated and expressed in the different light regimes (Table S8). We found only 23 TEs that were differentially methylated and differentially expressed across light regimes. Changes in frequency of light (SQH vs FLH and SQL vs FLL) accounted for the majority of these TEs, 12 and 7 respectively. Under SQH vs FLH, 10 of the 12 TEs showed an increase in expression, suggesting increased TE transcription under SQH light compared with FLH light (of which four displayed loss of DNA methylation; ATIS11A, ATGP3, ATLANTYS1, and VANDAL20) and two a loss of expression (of which one displayed gain of methylation; TA11). By contrast, under SQL vs FLL, six of the seven TEs displayed a decrease in expression (of which three displayed gain of DNA methylation; ATGP1, TA11, and a hAT‐like TE), with FLL light showing an increase in transcription of these TEs, and one TE displayed an increase in expression (coupled with a loss of DNA methylation; ATRE2).

**Fig. 5:**
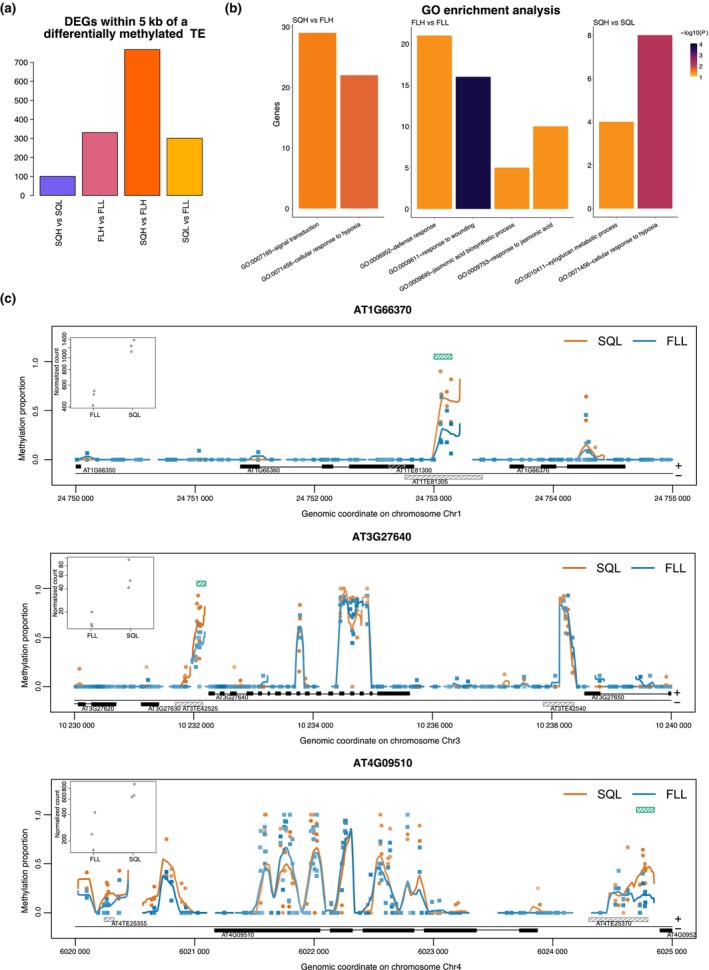
Differentially methylated transposable elements (TEs) have the potential to act as *cis*‐regulatory elements in *Arabidopsis thaliana*. (a) Overlap of differentially methylated TEs with differentially expressed genes (DEGs) within 5 kb. Approximately 29% of differentially methylated TEs between SQH and FLH, 14% of differentially methylated TEs between FLH and FLL, 12% differentially methylated TEs between SQL and FLL and 4% differentially methylated TEs between SQH and SQL are linked to a nearby differentially expressed gene. (b) Gene ontology analysis revealed significant enrichment‐ under SQH vs SQL, FLH vs FLL, and SQH vs FLH. Bar colour shows the −log_10_(*P*‐value) (c) Local methylation profiles and gene expression data for three differentially methylated TEs with a differentially expressed gene within 5 Kb under SQL vs FLL. Inlays show the expression data for the identified gene. The green box represents the DMR in CpG context. SQH, SQL – square light regime of high or low intensity, respectively. FLH, FLL – fluctuating light regime of high or low intensity, respectively.

TEs can also act as regulatory regions (Ito *et al*., 2011; Deneweth *et al*., 2022). To assess the possible impacts of changes in TE DNA methylation levels on nearby gene expression, we also investigated the expression of genes within 5 kb of differentially methylated TEs (Table S5). Between 2083 (FLH vs FLL) and 2243 (SQL vs FLL), TEs had a differentially expressed gene within 5 kb. Interestingly, we found a strong link between changes in methylation at TEs and changes in gene expression at nearby genes, with up to 767 DEGs being associated with a nearby differentially methylated TE (Fig. 5a). In particular, SQH vs SQL showed a total of 101 DEGs associated with a nearby differentially methylated TE, while FLH vs FLL had 331. In the square vs fluctuating comparisons, SQH vs FLH displayed the greatest number of DEGs (767), while SQL vs FLL yielded 301 genes (Fig. 5a). GO analysis revealed significant enrichment across several terms under SQH vs SQL, FLH vs FLL, and SQH vs FLH (Fig. 5b). The cellular response to hypoxia was common to SQH vs SQL and SQH vs FLH, indicating that oxygen availability may be impacted under these regimes. Under FLH vs FLL, terms associated with defence and wounding were enriched, suggesting that the intensity of fluctuations may impact biotic stress responses. However, when the expression level of DEGs within 5 kb of a TE was plotted against the methylation level of the given TE, no significant correlation was noted, suggesting that the level of methylation may not be reflective of possible regulation of nearby genes (Fig. S8). The correlation did improve when considering a smaller distance (1 and 2 kb), but still no significant relationship was revealed (Fig. S8; Table S9). When the GO was updated for TEs within 2 kb, only defence responses in FLH vs FLL and signal transduction in SQH vs FLH remained enriched. Fig. 5(c) shows individual examples of differentially methylated TEs nearby promoters or in downstream regions of DEGs, indicating a functional role in some of these cases. A systematic analysis identified concordant patterns between expression and methylation (hypomethylated TE with increased expression or hypermethylated TE with decreased gene expression), with 207 and 146 displaying this pattern in SQH vs FLH and SQL vs FLL, respectively (Table S10).

Chromatin state is also known to influence the expression of genes, with TEs normally associated with tightly compacted heterochromatin, while genes are often found in looser, euchromatic regions (Lippman *et al*., 2004). To determine whether differentially methylated TEs are found in euchromatic or heterochromatic regions, we overlapped our data with publicly available chromatin state data (Jamge *et al*., 2023). First, we plotted the methylation proportion of differentially methylated TEs across each sequence context and four chromatin states (euchromatic, facultative heterochromatin, constitutive heterochromatin, and intergenic regions), allowing us to see whether changes in methylation were associated with any particular chromatin state (Fig. S9). In euchromatic regions, no significant differences in CG methylation were seen across regime comparisons (Fig. S9A). For CHG methylation, significant differences were found between SQHvSQL vs FLHvFLL and SQHvSQL vs SQLvFLL. For CHH, significant differences were observed between SQHvSQL vs SQLvFLL, and FLHvFLL vs both SQvFL comparisons, with SQLvFLL showing a greater proportion difference than all other comparisons, suggesting some impact of light intensity vs light regime.

In constitutive heterochromatin (Fig. S9B), significant differences in CG methylation were seen across all regimes, with both HLvLL regimes showing a gain in methylation, while SQvFL shows a loss. Generally, a gain of CHG methylation was seen across light regime comparisons. CHH methylation was lost in SQHvSQL and SQHvFLH but gained under FLHvFLL and SQLvFLL in constitutive heterochromatic regions, suggesting different impacts of light intensity and regime in these regions. Similar patterns were noted in facultative heterochromatic TEs and those mapping to intergenic regions (Fig. S9C,D).

Due to these differences, we next examined the correlation between DEGs within 5 kb of a differentially methylated TE, separated by chromatin context (Fig. S10). For euchromatin, we found no DEGs within 5 kb of a euchromatic TE in SQHvSQL, but a negative correlation was found across all other regimes, although none were significant (*P*‐value > 0.1). For TEs in facultative heterochromatin, a significant positive correlation (*P*‐value = 0.013) was found in SQHvSQL. Across all other regimes and chromatin states, no significant correlations were found (*P*‐value > 0.1), suggesting a limited impact of differentially methylated TE chromatin state and nearby gene expression.

Finally, we investigated whether TE class impacted the correlation between TE methylation and nearby gene expression (Fig. S11). TEs were grouped into DNA, Helitron, LINE, long terminal repeats (LTR), RathE, and Unassigned. No significant correlation was found across classes in SQHvSQL or SQLvFLL. However, under FLHvFLL, there was a significant, positive correlation (*P*‐value = 0.012) between the log2 fold change and methylation of ‘Unassigned’ TEs. Under SQHvFLH, LTR TE methylation was negatively correlated with gene expression (*P*‐value = 0.02), suggesting a potential relationship between high‐intensity FL, DNA methylation at LTRs, and regulation of gene expression.

### Loss of DNA methylation improves photosystem II efficiency under fluctuating light

**Fig. 6:**
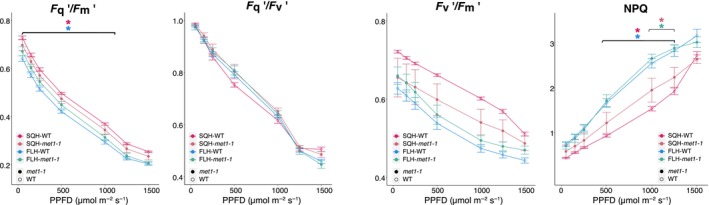
Chl fluorescence imaging of met1‐1 and wild‐type *Arabidopsis thaliana* acclimated to SQH and FLH in response to changing light. We considered (i) the operating efficiency of photosystem II (PSII) ($F_{q}^{\prime }/F_{m}^{\prime }$), (ii) photochemical quenching ($F_{q}^{\prime }/F_{v}^{\prime }$), (iii) maximum efficiency of PSII ($F_{v}^{\prime }/F_{m}^{\prime }$), and (iv) nonphotochemical quenching. *n* = 6, points represent the mean ± SE. Stars show the significant differences (*P* < 0.05; ANOVA and *post‐hoc* Tukey HSD test). SQH square light regime of high or low intensity, respectively. FLH, FLL – fluctuating light regime of low intensity.

To further investigate the link between DNA methylation and FL acclimation, we performed Chl fluorescence imaging on *met1‐1* knockdown plants following 7 d of acclimation to SQH or FLH regimes. *met1‐1* plants display *c*. 75% loss of CpG methylation, specifically in gene bodies (Kankel *et al*., 2003). This allows us to investigate which differences in physiological response under different light regimes require DNA methylation (in CpG context) to be established and maintained. Interestingly, we observed a difference between the WT and *met1‐1* plants under both regimes, with reduced methylation improving the PSII operating efficiency ($F_{q}^{\prime }/F_{m}^{\prime }$; Fig. 6) when acclimated to FLH light, with the opposite impact under SQH, although this was not significant. We also found a significant difference (*P < 0*.05) between SQH and FLH WT plants that was not identified in the *met1‐1* mutants (Fig. 6a,d). This appeared to be due to the $F_{v}^{\prime }/F_{m}^{\prime }$ rather than $F_{q}^{\prime }/F_{v}^{\prime }$, with decreases noted in the mutant relative to the WT under SQH, and a small increase in FLH‐acclimated *met1‐1* compared with WT (Fig. 6b,c). This suggests changes in the overall efficiency of PSII rather than a change in photochemical quenching, indicating CG methylation may contribute to regulating PSII efficiency under different light conditions. Overall, our results indicate that at least some of the changes in plant physiology (and, consequently, in gene expression) under SQ conditions do require DNA methylation. We also found a potential trade‐off between photosynthetic efficiency and acclimation under FLH conditions. To assess whether the observed differences could be due to common DMRs or DEGs between *met1‐1* and SQH vs FLH, we compared our data to previously published *met1‐1* data (Catoni *et al*., 2017). Over half of the CpG DMRs in SQH vs FLH were also differentially methylated between Col‐0 and *met1‐1*, with proportionally fewer shared in the CpHpG and CpHpH context (Fig. S12). Nevertheless, this is expected given that the *met1‐1* mutant mainly affects CpG methylation. All observed overlaps in DMRs and DEGs were found to be statistically significant (*P* < 0.05). Furthermore, of the 4635 in SQH vs FLH, 300 were also differentially expressed in the *met1‐1* mutant and only 17 of these displayed differential methylation in SQH vs FLH, including MCCA.

## Discussion

DNA methylation is highly dynamic and known to respond to environmental stimuli. There is limited evidence for the effects of long‐term light stress and acclimation on the methylome, with previous studies concluding there is little impact of light regime on DNA methylation despite clear physiological phenotypes (Ganguly *et al*., 2018). However, the impacts of natural light regimes and acclimation over the lifespan of *Arabidopsis* have not been conducted.

The peaks and troughs of the FL regimes are likely to act as cues for epigenetic change. For example, during the peaks of light in both FLH and FLL regimes, ROS are likely to be generated (Kono & Terashima, 2014). ROS generation has previously been associated with epigenetic change, and a recent study has correlated increased ROS due to abiotic stress with hypomethylation (Jing *et al*., 2022). Troughs may act as a recovery period for the plants, in which ROS production is reduced, and so could result in remethylation of the loci demethylated during high ROS. However, low light could also be considered as stressful for plants, prolonging vegetative growth (Xu *et al*., 2021) and reducing carbon assimilation capabilities (Vialet‐Chabrand *et al*., 2017), so it could itself cause hypomethylation. It is also important to note that, due to the LED system used, there are possible colour‐spectra differences at different light intensities, meaning the overall light spectra may be impacted in the fluctuating conditions used (Vong *et al*., 2025). In nature, spectral changes occur over the diurnal light cycle, with dominant blue wavelengths at dawn and dusk, and red peaking around midday (Kotilainen *et al*., 2020; Pastilha & Hurlbert, 2022). Therefore, these spectral artefacts could be considered as reflective of natural fluctuations experienced by plants, but further investigation is required to understand how spectral changes may impact these results.

There is increasing evidence for the impacts of light on plant epigenetic profiles with direct impacts on gene expression. For example, the histone demethylase INCREASE IN BONSAI METHYLATION 1 (IBM1), which removes methyl groups from H3K9 and acts to reduce non‐CpG methylation, has been found to impact anthocyanin biosynthesis in response to high light (Fan *et al*., 2024). Following 48 h of high light, IBM1 expression was induced in WT *Arabidopsis*, acting to increase the expression of SUPPRESSOR OF PHYA‐105 (SPA), which positively regulates the production of anthocyanin biosynthesis (Fan *et al*., 2024). This was attributed to both demethylation of H3K9 and to local decreases in non‐CpG methylation of the SPA genes (Fan *et al*., 2024). While there was no noted change in expression in IBM1 between light regimes, this provides evidence for direct epigenetic mechanisms associated with light exposure. Furthermore, more condensed chromatin has been associated with growth under light compared with dark conditions in soybean, with regulation of genes associated with photomorphogenesis particularly upregulated (Li *et al*., 2024). Together with our data, this suggests light conditions can specifically impact epigenetics and gene expression. Due to the unclear nature of the causality and correlation between DNA methylation and gene expression, it is difficult to conclude what the impact of light on methylation and gene expression truly are (Muyle *et al*., 2022; Grant *et al*., 2024).

Additionally, it is possible that the light regimes are impacting developmental timing and maturation. Under drought stress, various studies have demonstrated that stress induces senescence in older leaves while limiting overall leaf size (Skirycz *et al*., 2010; Clauw *et al*., 2016), linked to changes in the transcriptional profile (Swift *et al*., 2025). Increasing evidence suggests that DNA methylation has a role in plant ageing, with more loss of methylation with increased age seen in tree species *Pinus tabuliformis* (Li *et al*., 2023) as well as *Arabidopsis* (Dai *et al*., 2024). If the light regimes here are acting as a stressor, it is possible that changes in both the transcriptomic profile and DNA methylation are contributing to changes in leaf developmental timing and overall plant maturation rate. This has possible implications for field‐grown crops, in which daily FL could be slowing growth and maturation.

Relatively few differentially methylated genes were found to be differentially expressed (Table S4) in this study. This is consistent with previous studies into the effects of stress. For example, only 9 of 1562 differentially methylated genes were differentially expressed under prolonged cold treatment in *Brassica rapa* (Liu *et al*., 2017), while only 31 of over 5000 DMRs had differential expression under iron deficiency in rice (Sun *et al*., 2021). Together, these studies demonstrate that the magnitude of differential methylation between treatments is often larger than the resulting differential expression, suggesting differential methylation is not always controlling expression. Similar findings were noted here, with only a small subset of DMRs and DEGs overlapping (Fig. 3d,e). Active transcription can lead to gene body methylation, in turn acting to regulate gene expression (Teixeira & Colot, 2009). However, the low overlap between DMRs and DEGs indicates this is not the case in our system.

We validated one of these genes, MCCA, and found that its knockout significantly impacted photosynthetic efficiency (Fig. 4e). MCCA is a subunit of mitochondrial protein 3‐methylcrotonyl‐coenzyme A carboxylase, involved in leucine catabolism (Che *et al*., 2002). There is limited evidence for the involvement of either MCCase or leucine in acclimation to the light regime, although the expression of MCCA has been shown to decrease under increasing light intensity (Che *et al*., 2002). The decrease in expression between SQ and FL light regimes (Fig. 4a,b), accompanied by an increase in NPQ (Fig. 4e) in the mutant under FLL, suggests that MCCA or the products of leucine degradation may have some role in light harvesting or photoprotection under FL conditions. The opposite trend was seen under SQL, with slightly lower NPQ in SQL‐*mcca* than in Col‐0. Overall, the knockout of MCCA not only improved photochemical quenching but also increased NPQ under FLL, so while knocking out the gene may improve photosynthetic capacity, it may also increase the proportion of energy that cannot be used. MCCA is also conserved in crops (including rice and wheat), which potentially makes this an avenue for further research in improving crop adaptation to changing light patterns. However, only one mutant line was investigated, meaning this observation requires further validation.

Interestingly, we observed a change in methylation status and expression at a range of TEs. Activation of TEs has been seen under heat stress in *Arabidopsis*, with novel insertions reported (Ito *et al*., 2011; Sanchez *et al*., 2017; Roquis *et al*., 2021). However, while activation of TEs is a possibility, it is more likely that TEs act as *cis*‐regulatory regions to nearby genes. We found that hundreds of genes within 5 Kb of a differentially methylated TE were differentially expressed in different light regimes. Increased methylation of TEs has previously been shown to impact neighbouring gene expression. For example, in *Arabidopsis*, silencing of a SINE TE neighbouring FLOWERING WAGENINGEN (FWA), a locus associated with flowering, results in FWA silencing (Kinoshita *et al*., 2007). Methylation has been noted to spread from TEs into neighbouring genes, and the repressive chromatin state resulting from this has been proposed to also impact nearby genes (Ahmed *et al*., 2011). It is also possible that loss of methylation leads to increased expression of TE‐neighbouring genes due to the opening of the chromatin structure. However, DNA methylation can also promote gene activation. Transcriptional antisilencers SUVH1 and SUVH3 bind directly to methylated DNA and act as recruitment platforms for transcriptional enhancers DNAJ1 and 2 (Harris *et al*., 2018), with SUVH1 also shown to promote expression of promoter‐methylated genes (Li *et al*., 2016), possibly accounting for the lack of correlation between methylation and transcription seen here. Since this mechanism is reliant on CHH methylation (Harris *et al*., 2018), usually associated with TE silencing, it could be that increased methylation of TEs near genes results in increased expression. We noted a significant positive correlation between ‘Unassigned’ family TE methylation and nearby gene expression (Fig. S10), suggesting this could be the case here and warranting further investigation. Our study demonstrates the importance of light regime (the pattern in which light changes) for plant growth and development. Comparisons of light patterns (SQ to FL) resulted in a greater level of both epigenetic and transcriptomic change in genes and TEs, indicating substantial differences in the way plants respond to dynamic light environments than to the intensity of the light (high compared to low). This could have significant impacts on studies aimed at field crops grown under laboratory conditions, as this provides evidence as to how and why plants may react differently in laboratory and field studies. Furthermore, our results indicate that acclimation to light patterns involves a greater level of epigenetic and transcriptomic changes than acclimation to light intensity, which is of relevance to field plants and current climate changes.

## Competing interests

None declared.

## Author contributions

RAE, TL, UB and NRZ conceived and designed the experiments. RAE performed the experiments and analysed the data. PD and MK performed experiments on the *mcca* mutants. TL, UB and NRZ supervised the work. RAE, TL and NRZ wrote the paper. The authors read and approved the final manuscript.

## Disclaimer

The New Phytologist Foundation remains neutral with regard to jurisdictional claims in maps and in any institutional affiliations.

## Supporting information


**Fig. S1** Diurnal light regimes utilized in this study alongside physiological assessment.
**Fig. S2** Effects of light regime on DNA methylation.
**Fig. S3** Number of differentially methylated regions in different comparisons.
**Fig. S4** RNA‐seq data in different light regimes.
**Fig. S5** Gene ontology of overlapping differentially expressed genes between light regime comparisons.
**Fig. S6** Genes that are differentially methylated at the promoter and are differentially expressed.
**Fig. S7** Heatmaps of diurnal expression of genes differentially methylated and expressed in square vs fluctuating and high‐ vs low‐light regimes
**Fig. S8** Correlation between changes in methylation at transposable elements and changes in expression of nearby genes.
**Fig. S9** Methylation proportion across cytosine contexts in transposable elements found in different chromatin states.
**Fig. S10** Correlation between differentially methylated transposable elements and expression of genes within 5 kb across light regime comparisons, separated by the chromatin context of the transposable element.
**Fig. S11** Correlation between differentially methylated transposable elements and gene expression within 5 kb separated by transposable element class for each light regime comparison.
**Fig. S12** Overlap between differentially methylated regions and differentially expressed genes in met1‐1 mutant and in square light high vs fluctuating light high.

**Table S1** Light regimes utilized in this study.
**Table S2** Statistics for preprocessing Whole‐Genome Bisulfite Sequencing data.
**Table S3** Statistics for preprocessing RNA‐seq data.
**Table S4** List of all differentially methylated regions.
**Table S5** List of light constitutive genes and transposable elements.
**Table S6** List of all differentially expressed genes.
**Table S7** Functions of genes that are both differentially methylated and differentially expressed in growth light regime comparisons.
**Table S8** List of differentially methylated and differentially expressed transposable elements.
**Table S9** Differentially expressed genes with 5 kb of a differentially methylated transposable element.
**Table S10** Concordant differentially expressed genes within 1, 2, and 5 kb of a differentially methylated transposable element.
Please note: Wiley is not responsible for the content or functionality of any Supporting Information supplied by the authors. Any queries (other than missing material) should be directed to the *New Phytologist* Central Office.

## Data Availability

All WGBS and RNA‐seq data sets from this study have been submitted to the NCBI Gene Expression Omnibus (GEO; http://www.ncbi.nlm.nih.gov/geo/) under accession no. GSE261533. Codes for analysis are available on GitHub: https://github.com/robynemm/Emmerson‐et‐al_FL‐DNAmet.
