# Supplementary material for: DNA methylation contributes to plant acclimation to naturally fluctuating light
Source: New Phytol. 2025 Sep 20;248(5):2361–75. doi: 10.1111/nph.70567 (PMC12589712; doi:10.1111/nph.70567)
Supplement: Supplementary file 1 — Fig. S1 Diurnal light regimes utilized in this study alongside physiological assessment. Fig. S2 Effects of light regime on DNA methylation. Fig. S3 Number of differentially methylated regions in different comparisons. Fig. S4 RNA‐seq data in different light regimes. Fig. S5 Gene ontology of overlapping differentially expressed genes between light regime comparisons. Fig. S6 Genes that are differentially methylated at the promoter and are differentially expressed. Fig. S7 Heatmaps of diurnal expression of genes differentially methylated and expressed in square vs fluctuating and high‐ vs low‐light regimes Fig. S8 Correlation between changes in methylation at transposable elements and changes in expression of nearby genes. Fig. S9 Methylation proportion across cytosine contexts in transposable elements found in different chromatin states. Fig. S10 Correlation between differentially methylated transposable elements and expression of genes within 5 kb across light regime comparisons, separated by the chromatin context of the transposable element. Fig. S11 Correlation between differentially methylated transposable elements and gene expression within 5 kb separated by transposable element class for each light regime comparison. Fig. S12 Overlap between differentially methylated regions and differentially expressed genes in met1‐1 mutant and in square light high vs fluctuating light high. [file NPH-248-2361-s001.pdf]

New Phytologist Supporting Information

Article title: DNA methylation contributes to plant acclimation to naturally fluctuating light

Authors: Robyn A Emmerson, Philip Davey, Mouesanao Kandjoze, Ulrike Bechtold, Nicolae Radu Zabet*, Tracy Lawson*

Article acceptance date: 27 August 2025

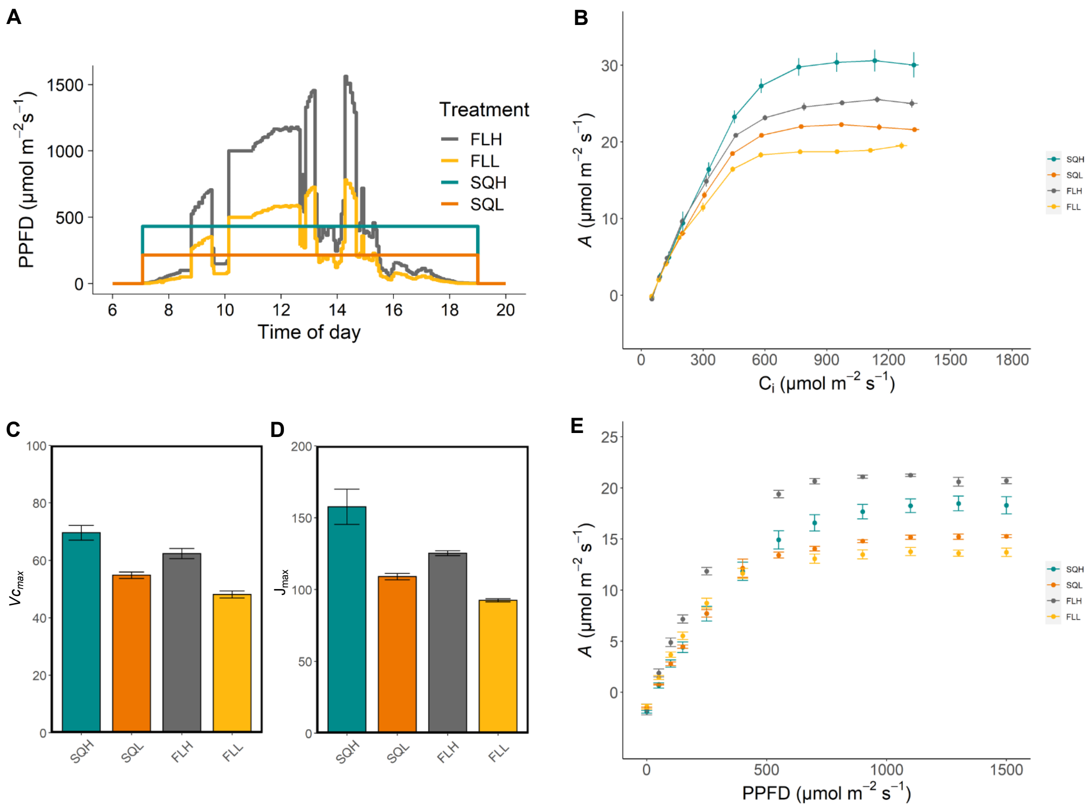

Figure S1: *Diurnal light regimes utilised in this study alongside physiological assessment. (A)* Area of the curves are equal, demonstrating the same average amount of light energy over a 12-hour period in low (square wave, SQL; fluctuating wave, FLL) and high light conditions (square wave, SQH; fluctuating wave, FLH). These calculations were originally performed by (Vialet-Chabrand *et al.* 2017) *(B)* Assimilation as a function of internal CO_2 concentrations in mature plants acclimated to each of the light regimes. From this, the maximum rate of carboxylation of Rubisco *(*V_{cmax}*; C)* which was significantly different across regimes, with both high light regimes having a greater rate than under low light, and fluctuating regimes performing worse than their square light counterpart ($p<0.05$). The maximum electron transport rate for RuBP regeneration *(*J_{max}*; D)* was also calculated*,* which demonstrated a significant decrease in RuBP regeneration in low light acclimated plants compared to high light plants ($p<0.05$). *(E)* The effect of changing Photosynthetic Photon Flux Density (PPFD; µmol m^{-2} s^{-1}) on photosynthesis in plants grown under the described light regimes. Letters on B and C indicate the results of Tukey post-hoc testing. Data shows the means ± SE (N=6 plants)

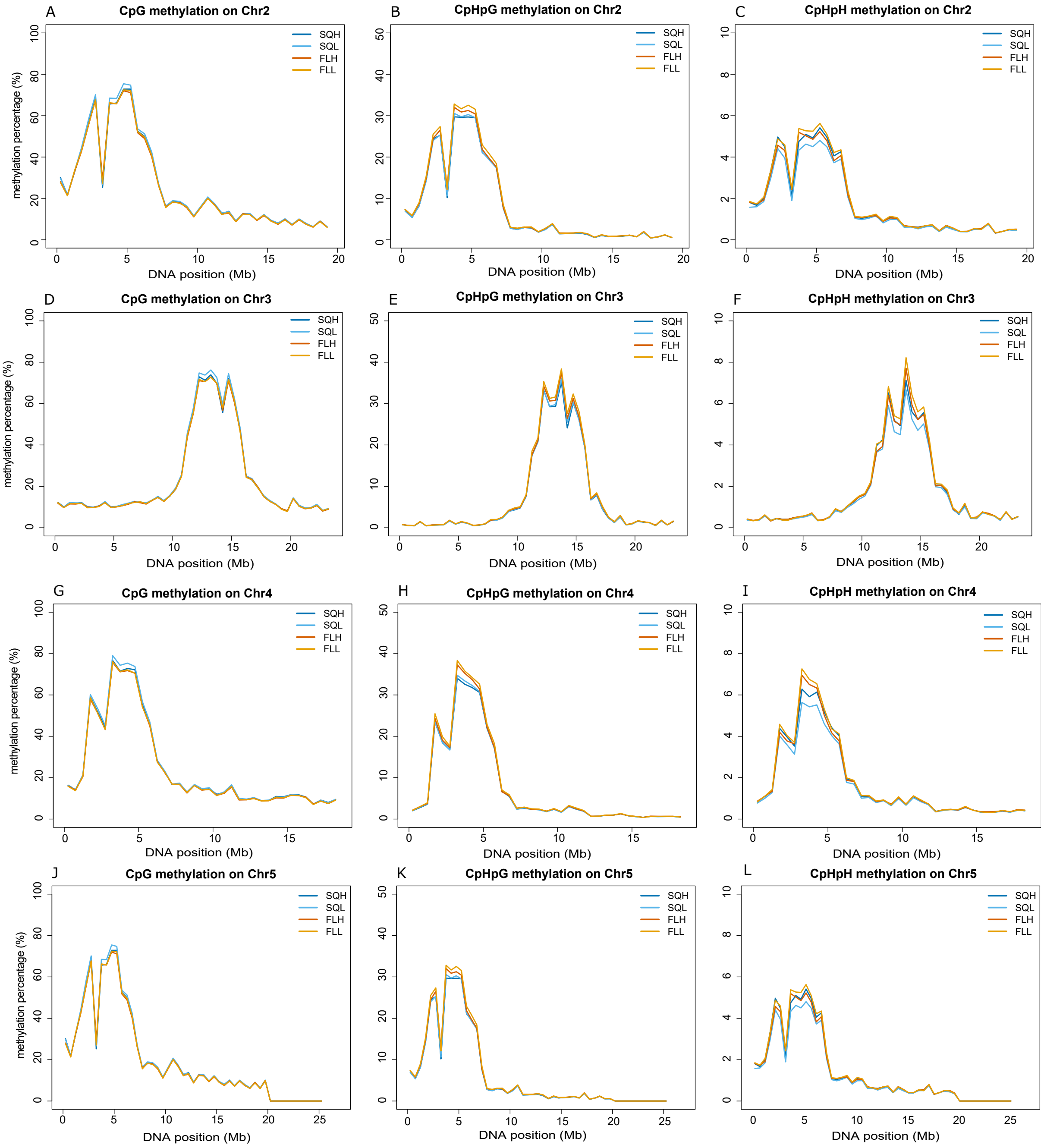

Figure S2: *The effects of light regime on DNA methylation*. Low resolution profiles of DNA methylation in the four light conditions. We considered separately CpG, CpHpG and CpHpH methylation patterns and plot methylation data on chromosomes 2 to 5.

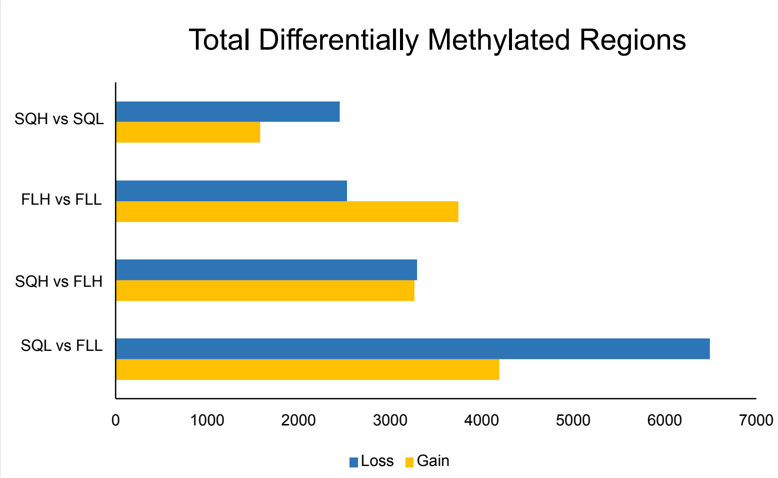

Figure S3: *Number of DMRs in different comparisons.* Bar plot represents the number of DMRs that loss (blue) or gained methylation in the second light regime compared to the first one. We considered the comparison of light intensity exposure (SQH vs SQL and FLH vs FLL) and of light frequency (SQH vs FLH and SQL vs FLL). The barplot includes all DMRs in CpG, CpHpG and CpHpH contexts.

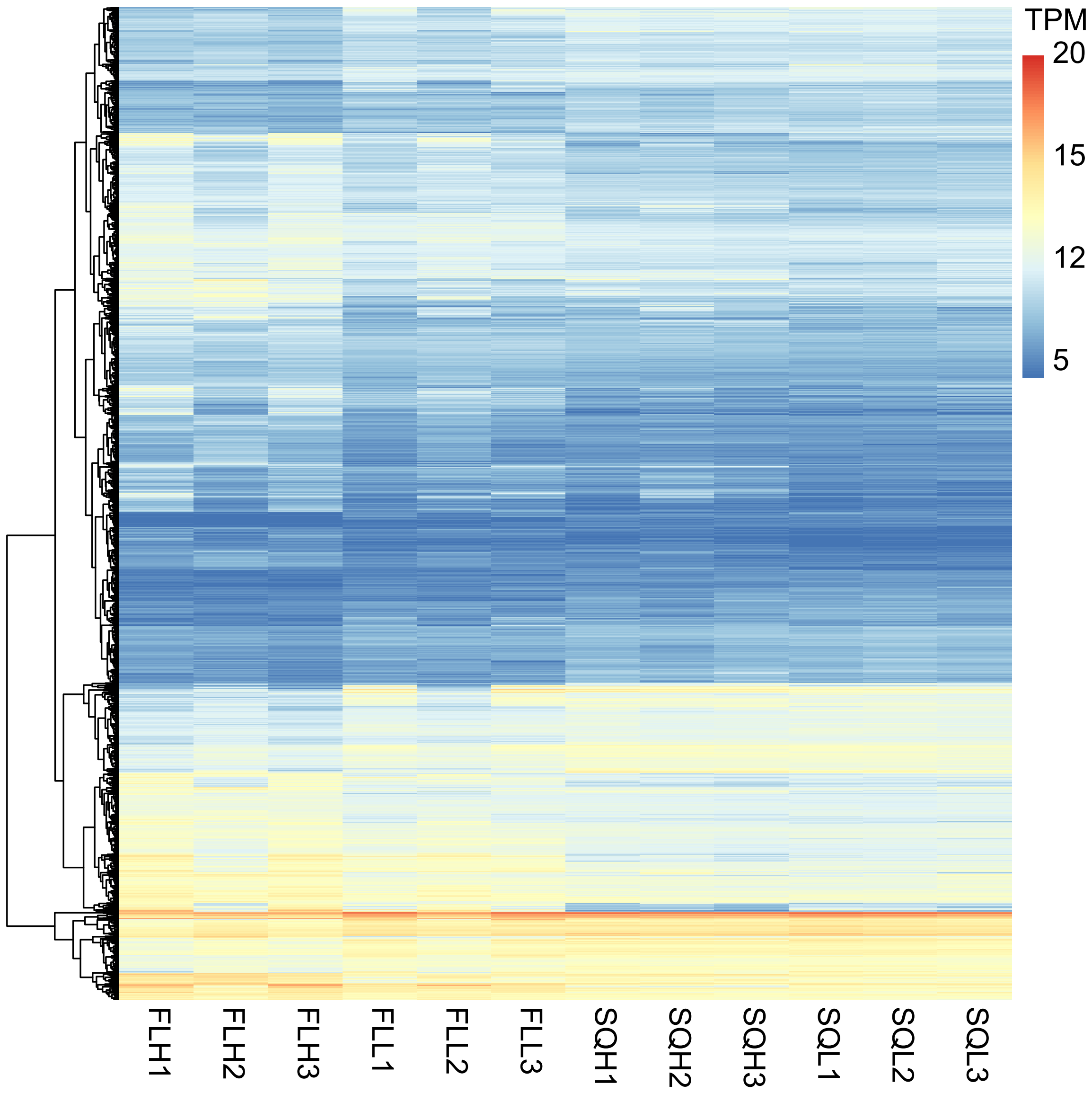

Figure S4: *RNA-seq data in different light regimes.* The heatmap shows transcripts per million (TPM) for each gene for each of the three replicates of the four light regimes.

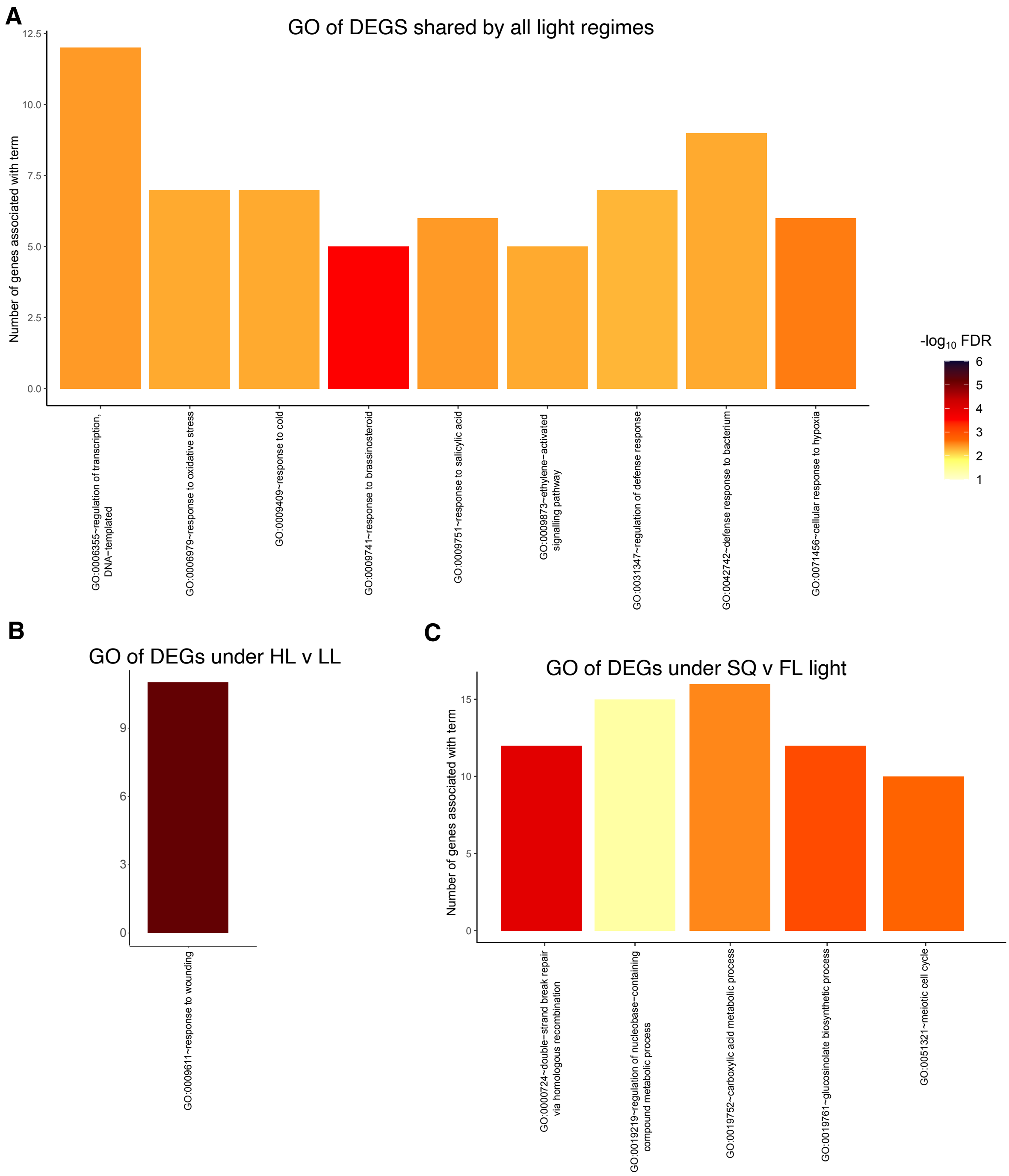

Figure S5: *Gene ontology of overlapping differentially expressed genes between light regime comparisons*. Bar height shows the number of genes associated with the indicated term, while colour indicated the -$\log_{10}$ of the False Discovery Rate (FDR).

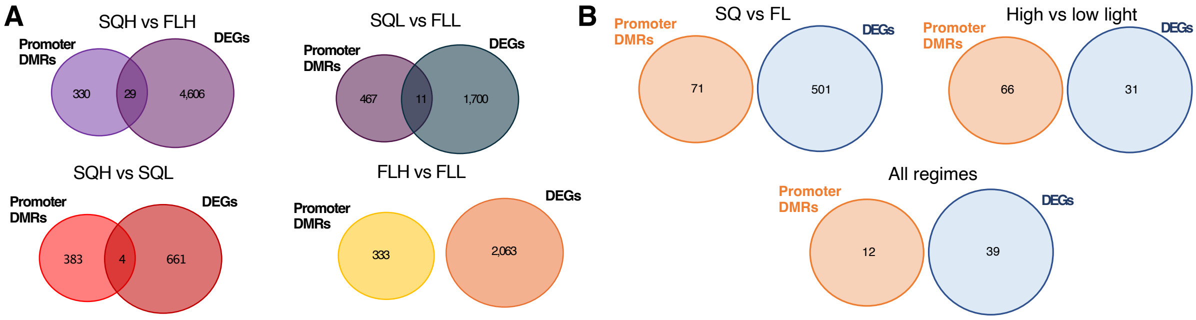

Figure S6: *Genes that have are differentially methylated at the promoter and are differentially expressed*. *(A)* Venn diagrams of genes that are differentially expressed in one of the four comparisons and also overlap with a DMR (in any context) within the promoter of the gene in the corresponding comparison. *(B)* Genes that are differentially expressed and overlap with DMRs within the promoter of the gene in the three combined comparisons, namely: *(i)* fluctuating vs square light, *(ii)* high vs low intensity and *(iii)* all comparisons.

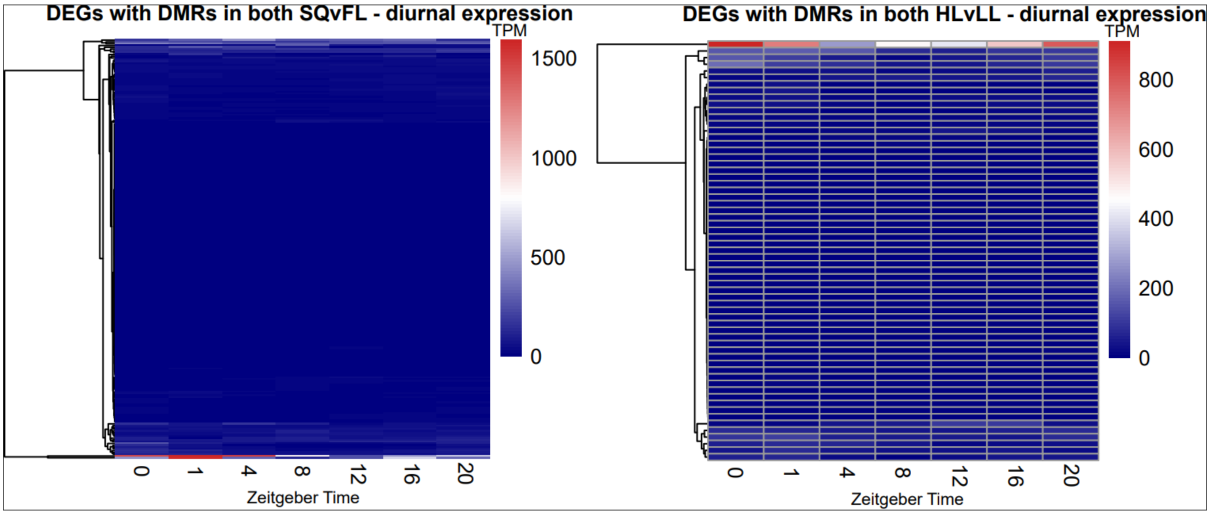

Figure S7: *Heatmaps of diurnal expression of genes differentially methylated and expressed in square vs fluctuating (SQvFL) and high vs low (HLvLL) light regimes*. Genes were overlapped with expression data from Redmond et al. (2025) and plotted over the time course. The heatmap shows transcripts per million (TPM)

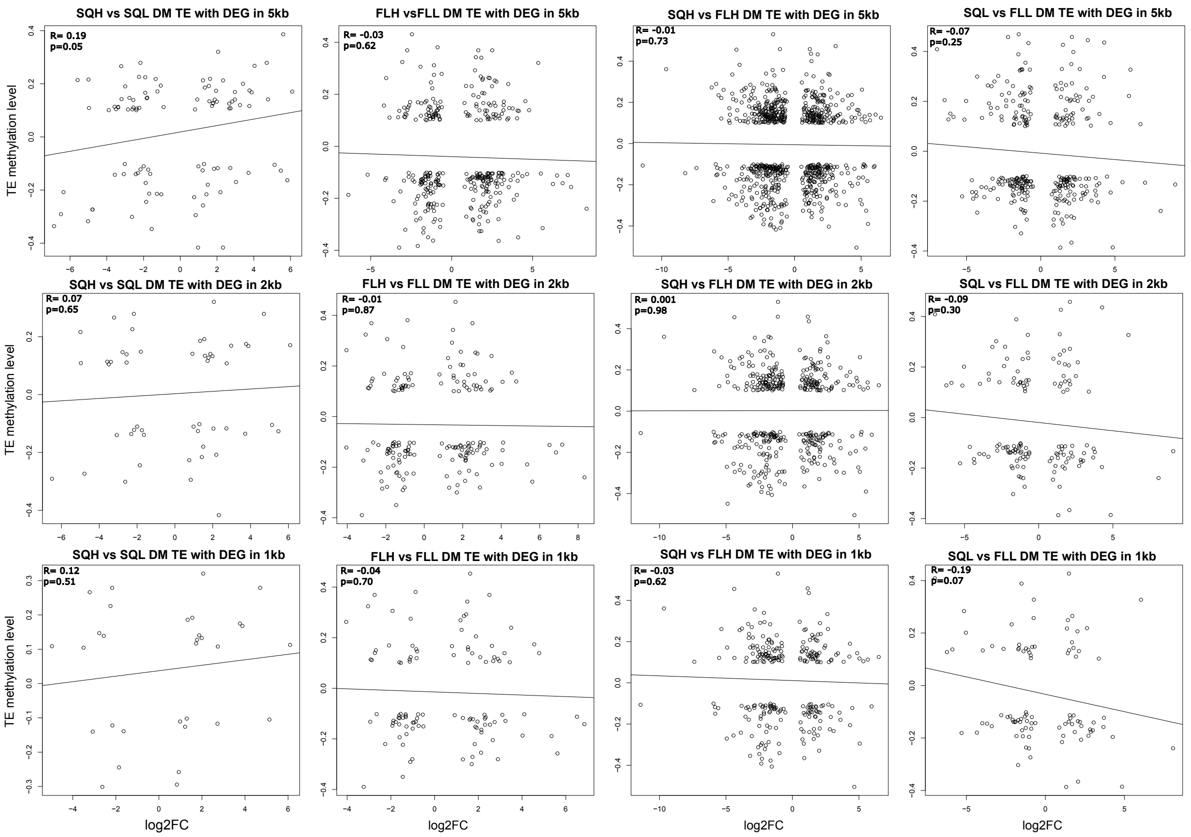

Figure S8: *The correlation between changes in methylation at transposable elements and changes in expression of nearby genes*. We considered separately the case of different distances between the TE element and the gene, namely: 1, 2, or 5 Kb. Only differentially expressed genes were included in this analysis.

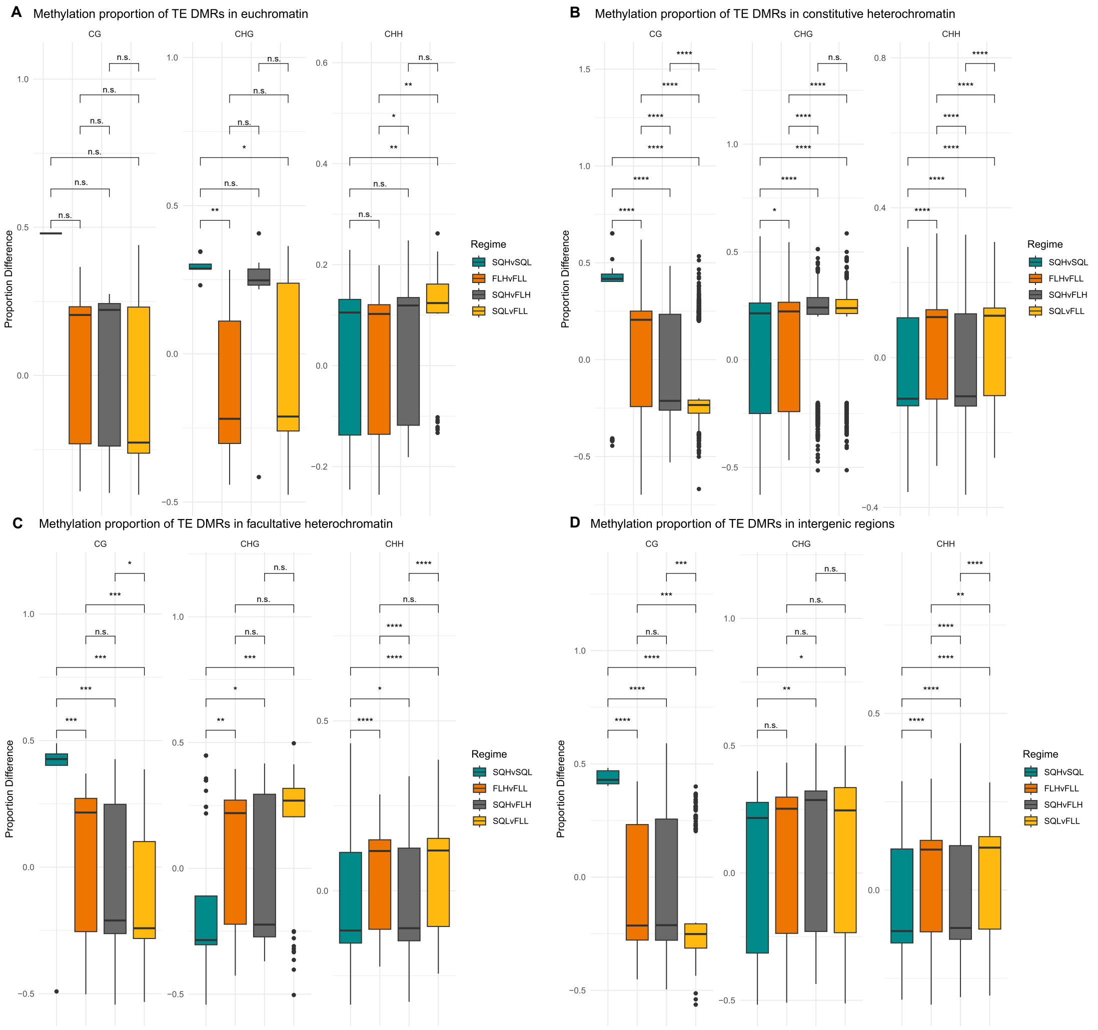

Figure S9: *Methylation proportion across cytosine contexts in transposable elements (TEs) found in different chromatin states*. *(A)* Euchromatin. *(B)* Constitutive heterochromatin*.* *(C)* Facultative heterochromatin. *(D)* Intergenic regions. We also performed the Mann–Whitney U test between the different conditions(p-value: n.s. ≥ 0.05, *p value < 0.05, ** < 0.01 and *** < 0.001).

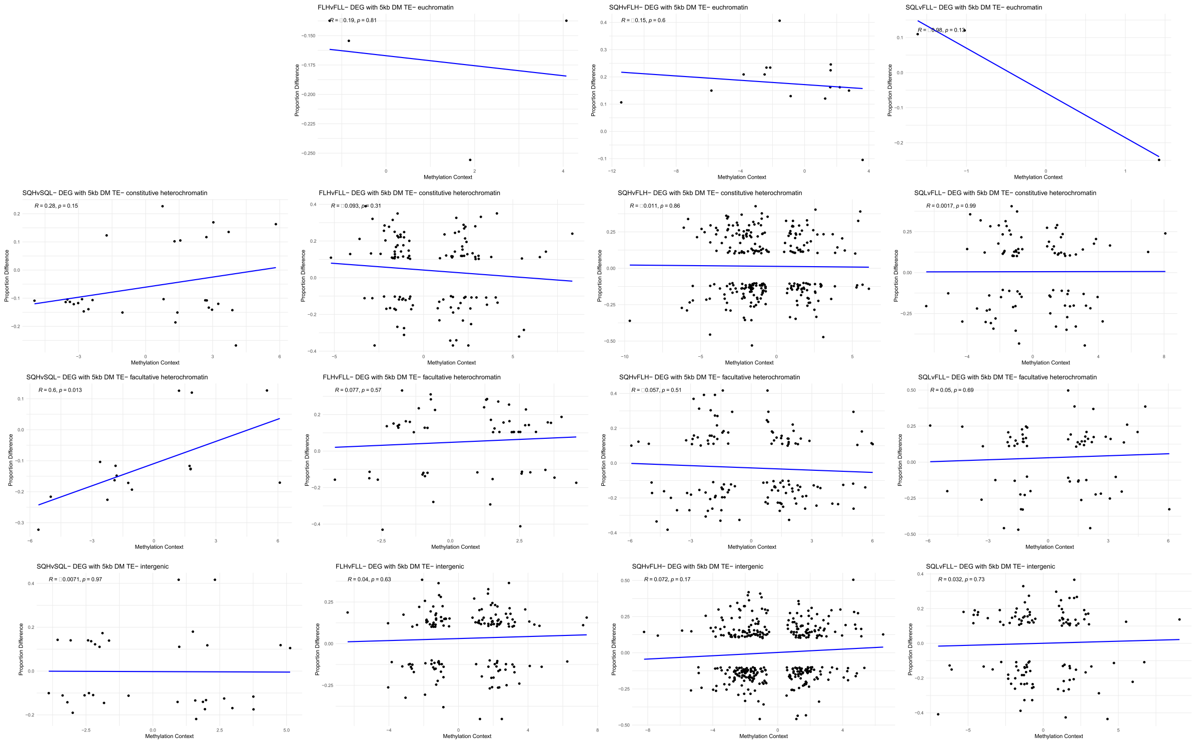

Figure S10: *Correlation between differentially methylated transposable elements (TE) and expression of genes within 5kb across light regime comparisons, separated by the chromatin context of the TE.* Only differentially expressed genes were included in this analysis.

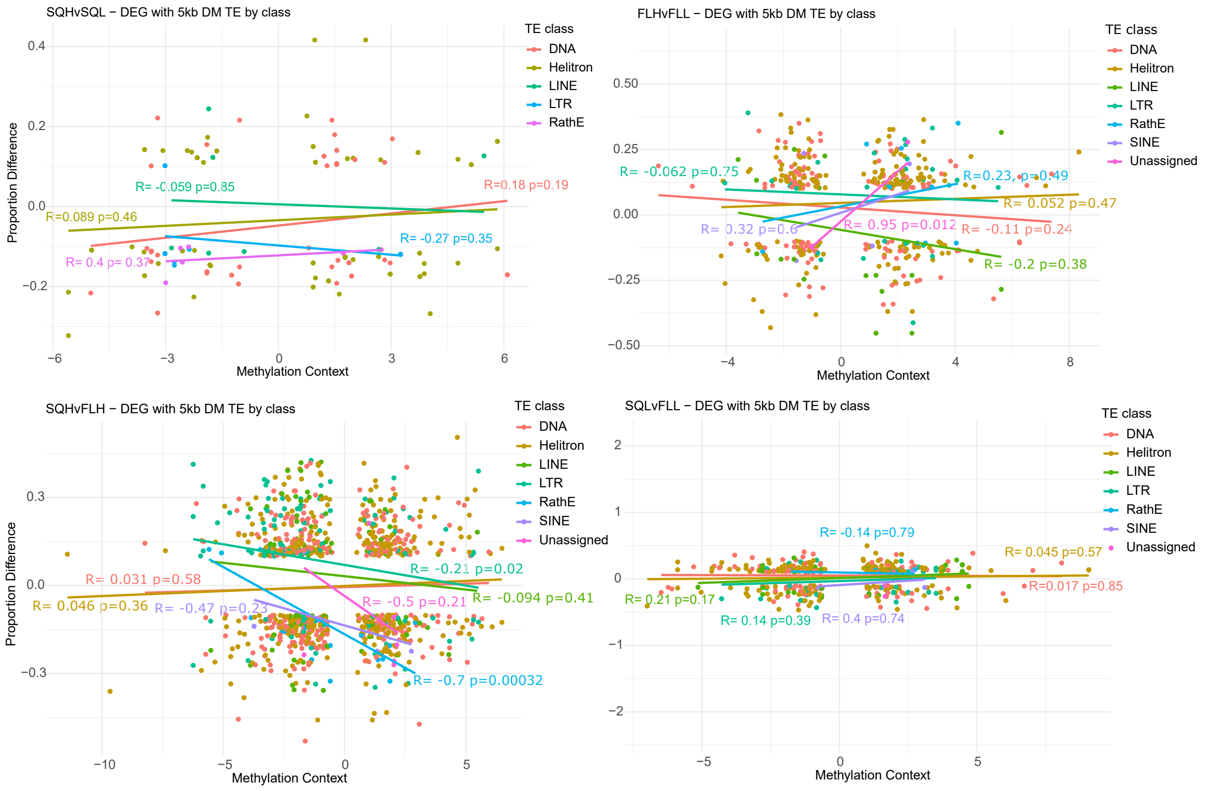

Figure S11: *Correlation between differentially methylated transposable elements (TEs) and gene expression within 5kb separated by TE class for each light regime comparison.* Only differentially expressed genes were included in this analysis.

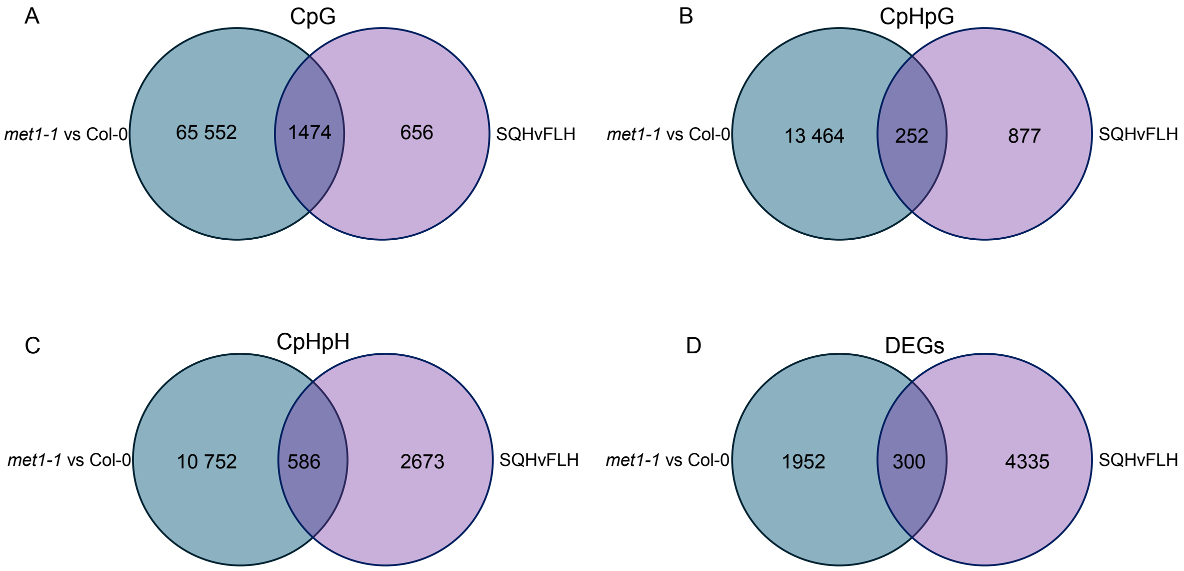

Figure S12: *Overlap between DMRs and DEGs in met1-1 mutant and in SQH vs FLH*. (A-C) Venn diagrams showing the overlap between DMRs in met1-1 vs Col-0 and SQH vs FLH light regimes. We considered the three contexts (CpG, CpHpG and CpHpH) separately. (D) Venn diagram showing the overlap between differentially expressed genes in met1-1 vs Col-0 and SQH vs FLH light regimes. For the DEGs in met1-1, only expression array data was available (Catoni et al., 2017), which could partially explain the lower overlap.
